# Applications of Membranes for Sustainability

**DOI:** 10.3390/membranes11080629

**Published:** 2021-08-16

**Authors:** Veeriah Jegatheesan, Chettiyappan Visvanathan, Li Shu, Faisal I. Hai, Ludovic F. Dumée

**Affiliations:** 1 Effective Technologies and Tools Research Centre, School of Engineering and Water, RMIT University, Melbourne, VIC 3000, Australia; 2 School of Environment, Resources and Development, Asian Institute of Technology, Klongluang, Pathumthani 12120, Thailand; visu@ait.ac.th; 3 LJS Environment, Parkville, VIC 3052, Australia; li.shu846@gmail.com; 4 School of Engineering, Edith Cowan University, Joondalup, WA 6027, Australia; 5 Strategic Water Infrastructure Laboratory, School of Civil, Mining and Environmental Engineering, University of Wollongong, Wollongong, NSW 2522, Australia; faisal@uow.edu.au; 6 Department of Chemical Engineering, Khalifa University, Abu Dhabi P.O. Box 127788, United Arab Emirates; ludovic.dumee@ku.ac.ae

Applications of membranes in water and wastewater treatment, desalination, as well as other purification processes, have become more widespread over the past few decades. However, we continue to ponder on (i) how well do we get maximum benefits from membrane applications? (ii) In what areas of membranes do we need to further our fundamental understanding? (iii) Which membrane technologies find difficulties in scale-up? (iv) How good are we in integrating membrane technologies? (v) How can we improve the reliability of membranes and reduce the cost? (vi) Where are we in disseminating the needs if we are to use membranes all over the world? (vii) How can we bring true global collaborations? The membrane community all over the world is continuing to contribute to answer those questions. This Special Issue is also contributing answers to some of the above questions. The call for papers for this Special Issue invited researchers broadly, and the delegates of the International Conference on the Challenges in Environmental Science and Engineering, CESE-2019 (3–7 November 2019, at the Grand Hi-Lai Hotel in Kaohsiung, Taiwan) in particular, who are working on the above to submit original research papers, critical review articles, case studies and technical notes.

Six articles investigating the performance of hybrid direct contact membrane distillation (DCMD)/UV photolysis and reverse osmosis (RO)/forward osmosis (FO) membranes, modification of membranes for photocatalysis and enhanced anti-bacterial properties, reviewing fouling of membrane bioreactors (MBRs) and predicting the fouling of MBRs are included in this collection. Tufail et al. [1] investigated the performance of hybrid DCMD and UV photolysis in degrading five trace organic contaminants (TrOCs). The study found that the nature and extent of the impact of inorganic ions, such as halides, nitrates and carbonates, as well as organic substance (humic acid), in degrading TrOCs depended on the type of TrOCs (phenolic contaminants such as bisphenol A and oxybenzone and non-phenolic contaminants such as sulfamethoxazole, carbamazepine, and diclofenac) and the concentration of the interfering ions. Thus, continued research is warranted to develop a database containing the performance of such a hybrid system in treating various TrOCs. Liyanaarachchi et al. [2] utilised FO to dilute RO concentrate, either to increase the water recovery from the RO system, or to discharge the diluted RO concentrate safely to the environment. They utilised the pre-treatment (sand filter) backwash water containing ferric hydroxide as the feed solution and RO concentrate as the draw solution for the FO process. The study found that cellulose triacetate (CTA) flat sheet FO membrane produced higher flux (3–6 L m^−2^ h^−1^) compared to that produced by polyamide (PA) hollow fibre FO membrane (less than 2.5 L m^−2^ h^−1^) under the same experimental conditions. Long-term studies conducted on the flat sheet FO membranes showed that fouling due to ferric hydroxide sludge did not allow the water flux to increase more than 3.15 L m^−2^ h^−1^. Increasing the water flux needs further investigation in order for the process to have practical applications.

Sakakar et al. [3] synthesised thin-film composite (TFC) polyvinylidene fluoride (PVDF) membranes by coating with titanium dioxide (TiO_2_)/polyvinyl alcohol (PVA) solution through the dip-coating method, and cross-linked it with glutaraldehyde to improve the thermal and chemical stability of the thin film coating. The study found that the layer of TiO_2_ nanoparticles on the PVDF membranes reduced the fouling effects compared to the plain PVDF membrane. The study also showed that nearly 78% methyl orange and 47% reactive blue dyes were removed by the TFC membrane, along with photodegradation. However, the membrane can be damaged by the radicals formed by TiO_2_ during UV irradiation. Further studies are required to improve the stability of the membrane by finding suitable polymers or using inorganic membranes which are less susceptible to the attack by the radicals. Samree et al. [4] modified the PVDF membrane by coating it with nanoparticles of titanium dioxide (TiO_2_-NP) and silver (Ag-NP) at different concentrations and coating times to improve the hydrophilicity and antibacterial properties of the membrane. The study compared both the plain and modified PVDF membrane with respect to their performance in reducing Escherichia coli cells and inhibiting the formation of biofilm on the membrane surface. Compared to plain PVDF membrane, the modified membrane exhibited antibacterial efficiency up to 94% against E. coli cells, and inhibition up to 65% of the biofilm mass reduction.

Du et al. [5] carried out an extensive review on the mechanisms, impacts and control methods of membrane fouling in MBR systems. Compared to conventional activated sludge process, an MBR has many advantages, such as good effluent quality, small floor space, low residual sludge yield and easy automatic controls. It also allows slow growing microbes to exist and therefore remove hardly degradable pollutants. The treated effluent by MBR will generally meet Class A standards and therefore can be reused in various applications. However, membrane fouling is the main obstacle to the wider application of MBR. The authors suggest focussing the future research on the following: (i) Exploring real-time formation of organic fouling of membranes to optimise periodic cleaning. (ii) Developing new membranes with increased anti-fouling properties; low energy consumption, less chemical usage, and easier operation and maintenance should also be researched. Comprehensive economic analysis, life cycle assessment, and carbon footprint analysis of different direct membrane filtration processes should be conducted in order to identify the most suitable system configuration for further scale-up. (iii) Finding hybrid methods with low energy consumption for improving the application of ultrasonic technology in full-scale MBR systems. (iv) Optimising the aeration; the method of aeration could be optimized according to computational fluid dynamic modeling on the fluidization and the scouring behavior of the particles in MBRs. Moreover, the attachment tendency of biofilm colonizers on the medium and membranes should be assessed. Shi et al. [6] explored the recent advances in predicting the fouling of MBRs. They reviewed the techniques available to predict fouling in MBRs and discussed the problems associated with predicting fouling status using artificial neural networks and mathematical models. The authors suggest that the following need further research: (i) Fouling mechanisms in MBRs of different structures and scales with the development of accurate and real-time online data on membrane fouling. (ii) Study on remaining useful life (RUL) predictions of the membrane modules at various failure modes, as failure of membrane modules is usually caused by the synergistic effect of multiple failure modes. (3) Intelligent feature extraction by deep learning, such as a deep belief network and convolutional neural network.

Thus, the articles provided in this Special Issue disseminate both the current knowledge available and future research needs in the respective topics discussed. We hope this collection will be useful for developing plans for future research topics on the applications of membranes for sustainability.
